# Arthroscopic-assisted versus fluoroscopic-assisted open reduction and internal fixation for distal radius fracture: A systematic review and meta-analysis

**DOI:** 10.1097/MD.0000000000041434

**Published:** 2025-02-07

**Authors:** Keyi Chen, Shun Yang, Yabo Cheng, Wang Xiang

**Affiliations:** a Department of Hand and Wrist Surgery, Sichuan Province Orthopaedic Hospital, Chengdu, Sichuan, China.

**Keywords:** arthroscopy, distal radius fracture, meta-analysis, ORIF

## Abstract

**Background:**

In recent years, there has been an increasing trend in the use of wrist arthroscopy to facilitate the treatment of distal radius fractures using open reduction and internal fixation (ORIF). However, there is no consensus on its superiority despite some previous studies comparing these 2 surgical techniques. Hence, this study sought to conduct a systematic comparison of the effectiveness and safety of arthroscopic-assisted open reduction and internal fixation (AAORIF) and fluoroscopic-assisted open reduction and internal fixation (FAORIF) in the management of distal radius fractures.

**Methods:**

A comprehensive search was conducted in multiple databases to acquire all literature published until January 31st, 2024, comparing AAORIF versus FAORIF for the treatment of distal radius fracture, without any language or publication status restrictions. The Cochrane Collaboration’s RevMan 5.4 software was used to perform a meta-analysis.

**Results:**

Five randomized controlled trials (169 patients in the AAORIF group and 171 patients in the FAORIF group) suggested that patients with distal radius fracture treated with AAORIF had better disability of arm, shoulder, and hand scores, visual analog scale score, and wrist extension than those treated with FAORIF. However, no significant differences were observed in terms of wrist flexion, pronation, supination, operative time, and complication outcomes between the 2 groups.

**Conclusion:**

Despite the lack of significant differences in wrist flexion, pronation, supination, operative time, and complication outcomes between the 2 groups, the AAORIF group had more obvious advantages in terms of disability of arm, shoulder, and hand scores, visual analog scale score, and wrist extension based on the above results. We believe that treatment of distal radius fracture with AAORIF may be an optimal option compared with FAORIF. Absolutely, and additional high-quality studies with larger sample sizes are necessary to establish stronger evidence regarding this subject.

## 1. Introduction

Distal radius fractures account for nearly 18% of all fractures in adults and are the reason for 1.5% of emergency room visits.^[1]^ About 10%of these fractures are intraarticular. Several studies have shown that a residual step-off or gap between fragments >2 mm or even 1 mm after fracture reduction can lead to radiocarpal osteoarthritis in up to 91% of cases at 6 years.^[2–6]^ Fluoroscopy, which is commonly used in the treatment of distal radius fractures, offers a more accessible and less complicated method for evaluating articular surface reduction.

Wrist arthroscopy has become increasingly popular in treating injuries to the distal radial articular surface and surrounding soft tissues of the wrist.^[7–10]^ This technique, first described in the 1990s, has been recognized as an effective adjunct for treating distal radius fractures. Arthroscopy offers a magnified view of the articular surface while being less invasive compared to arthrotomy. On the flip side, arthroscopically-assisted surgery provides the opportunity for more precise reduction of the articular surface and addressing soft-tissue injuries in complex intraarticular fractures of the distal radius.^[11–14]^ In theory, arthroscopy offers more benefits than fluoroscopy for treating distal radius fractures. However, certain scholars have discovered that fluoroscopic reduction using a high-quality image intensifier is equivalent to arthroscopic reduction.^[10]^

Whether FAORIF offers comparable functional and radiographic outcomes with arthroscopic-assisted open reduction and internal fixation (AAORIF) for the treatment of intraarticular fractures of the distal radius, no consistent conclusions have been reached regarding the superiority of one over the other.^[15–18]^ Currently, there are no well-conducted studies that compare the efficacy of arthroscopically assisted and fluoroscopically assisted techniques in reducing distal radius fractures. We conducted a meta-analysis that included only randomized controlled trials (RCTs) in order to compare the effectiveness and safety of the 2 surgical methods for treating distal radius fractures.

## 2. Materials and methods

### 2.1. Search strategy

We searched the online databases of PubMed, EMBASE, Web of Science, Cochrane Library, China National Knowledge Infrastructure Database, China Science and Technology Journal Database, Wanfang Database up to January 31, 2024 for studies that compared AAORIF versus FAORIF for treatment of distal radius fracture. The search strategy employed was as follows (distal radius fracture) and (arthroscopic-assisted ORIF or arthroscopic-assisted open reduction and internal fixation) and (fluoroscopic-assisted ORIF or fluoroscopic-assisted open reduction and internal fixation). There were no limitations on the timing of publication.

### 2.2. Inclusion criteria

The inclusion criteria were as follows: (1) patients diagnosed with distal radius fracture; (2) original studies directly comparing AAORIF with FAORIF to treat distal radius fracture and reporting at least one of the following outcomes: wrist extension-flexion, pronation-supination, disability of arm, shoulder, and hand (DASH) scores, visual analog scale (VAS) score, operative time, and complications; (3) study type: only RCTs; (4) language limited to English or Chinese (Table 1).

**Table 1:** Study characteristics.

Author	Year	Study design	Pt no (n)		Fracture type	Females n (%)		Age; mean ± SD		Study language	Risk of bias	Follow-up M	
			FAORIF	AAORIF		FAORIF	AAORIF	FAORIF	AAORIF			FAORIF	AAORIF
Dou^[19]^	2019	RCT	30	30	AO Type C	52.1	48.1	48.4	52.1	Chinese	Low	13	16
Selles^[20]^	2020	RCT	25	25	AO Type C	49.4	62.9	58.2	48.3	English	Low	23	19
Varitimidis^[21]^	2008	RCT	20	20	AO Type C	53.9	55.2	45.3	50.2	English	Low	20.	18
Yamazaki^[22]^	2015	RCT	34	36	AO Type C	67.1	62.3	62.4	60.1	English	Low	22	21
Yang^[23]^	2020	RCT	60	60	AO Type B and C	63.5	55.6	52.4	56.3	Chinese	Low	19	23

RCTs = randomized controlled trials.

### 2.3. Exclusion criteria

Studies that met the following criteria were excluded: (1) patients aged < 18 years or >70 years; (2) patients with a history of wrist trauma, multiple fractures in the wrist, chronic skin lesions, or any combination of infection, deformity, tumor, or rheumatoid arthritis; (3) studies that lacked valid data; (4) duplicate studies, conference abstracts, review articles, case reports, biomechanical studies, and cadaveric studies.

### 2.4. Data extraction and management

Two researchers conducted an independent review of the chosen publications, documenting the first author’s name, publication year, article language, country, average patient age, other characteristics, study design, and the number of patients receiving AAORIF and FAORIF treatment for distal radius fractures. The outcomes pooled in this analysis included operative time, range of motion (ROM) of wrist extension, flexion, pronation and supination, VAS score, DASH scores, and complications; any disagreements were resolved by a third reviewer.

### 2.5. Risk of bias assessment

Two reviewers estimated the quality of the included studies, and the Cochrane Back Review Group was used to estimate RCTs (Table 2)^[24]^. According to the number of conditions met by the 11 criteria, the included study was regarded as having a low risk of bias (RoB) or high RoB. All studies were classified as having minimal risk of selection bias because of the particularity of surgical selection; the performance bias was rated as having a high risk because blinding was not possible; if heterogeneity was found, the origins were examined based on variations in methodological quality, participant characteristics, and interventions.

**Table 2:** Methodologic quality assessment.

Risk of bias assessment of the randomized studies by the Cochrane Back Review Group (CBRG).
A. Was the method of randomization adequate? B. Was the treatment allocation concealed? C. Were the groups similar at baseline regarding the most important prognostic factors? D. Was the patient blinded to the intervention? E. Was the care provider blinded to the intervention? F. Was the outcome assessor blinded to the intervention? G. Were co-interventions avoided or similar? H. Was adherence acceptable in all groups? I. Was the dropout rate described and acceptable? J. Was the timing of the outcome assessment in all groups similar? K. Did the analysis include an intention-to-treat analysis?

### 2.6. Statistical analysis

The statistical analysis was conducted using the RevMan 5.4 software from Cochrane IMS. If dates were missed from published reports, we tried to contact corresponding authors for original data via email. The obtained data presented odds ratio and 95% confidence interval (95% CI) for dichotomous outcomes, and mean difference (MD) and 95% CI for continuous outcomes. Standardized mean difference and 95% CI were calculated when the same continuous outcomes were measured in different scales. The *I*^2^ statistic was used to evaluate heterogeneity. If the value of *I*^2^ is >50%, the random-effects model was used. Both a subgroup analysis and a sensitivity analysis were conducted to examine the source of heterogeneity. To conduct sensitivity analysis, articles with high statistical heterogeneity were excluded.^[25]^ Conversely, the fixed-effects model was applied, with *P* < .05, indicating statistical difference.

## 3. Results

### 3.1. Search results

**Figure 1.**
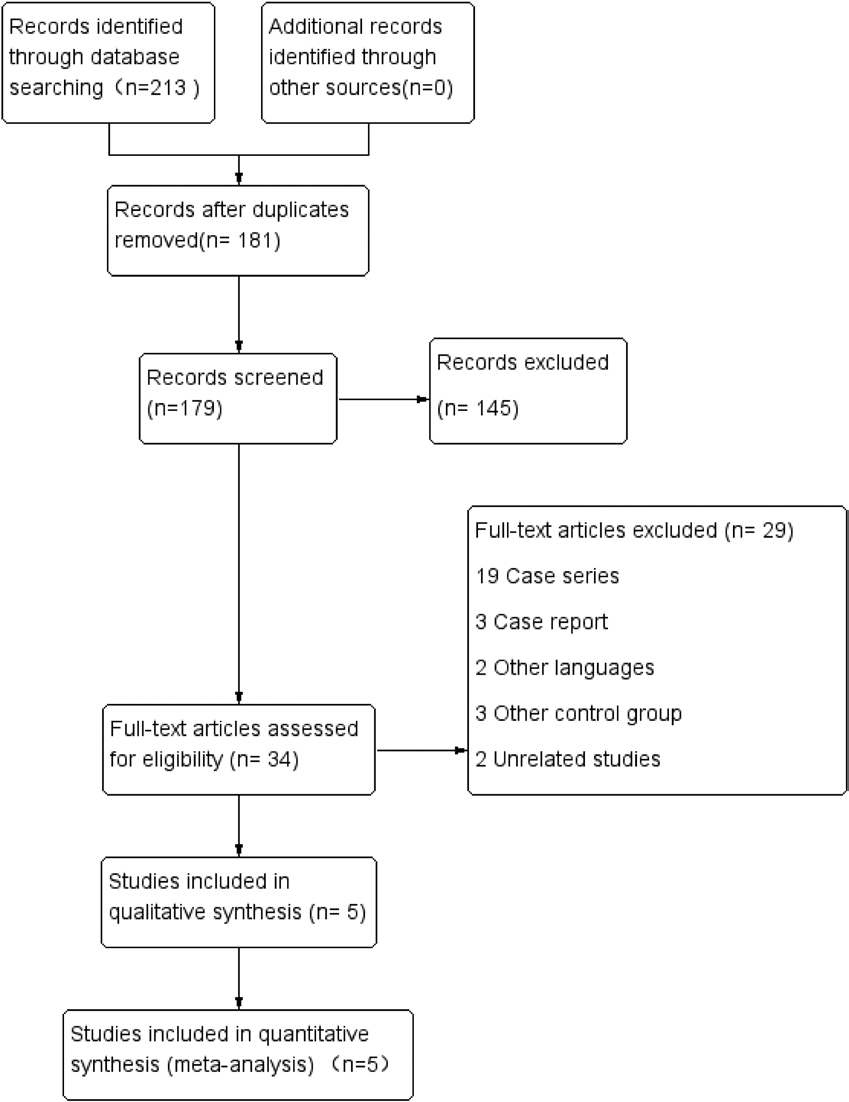
Study selection flowchart.

The initial search process and the relevant results are shown in Figure 1. After conducting the initial database search, a total of 213 publications were identified: PubMed yielded 55 publications, EMBASE yielded 37 publications, Web of Science yielded 52 publications, Cochrane library yielded 4 publications, China National Knowledge Infrastructure Database yielded 31 publications, China Science and Technology Journal Database yielded 13 publications, and Wanfang Data yielded 21 publications. Following the screening of titles and abstracts, 179 studies were excluded; we read the full texts of the remaining 34 studies and ultimately included 5 RCTs studies that fulfilled all eligibility criteria in this meta-analysis.

### 3.2. Study characteristics

The 5 studies^[19–23]^ were from China, Netherlands, Japan, and Greece, respectively. There were 3 articles written in English and 2 written in Chinese, and all of them were RCTs. There were a total of 340 patients across the 5 studies, with 169 patients in the AAORIF group and 171 patients in the FAORIF group. Table 1 displays the characteristics of the studies included and their RoB. Study quality assessment regarding methodological quality according to the Cochrane Back Review Group,^[24]^ 5 RCTs had a low RoB with a score of 6 to 8. Overall, this study has a low RoB.

### 3.3. Meta-analysis results

#### 3.3.1. Flexion

**Figure 2.**
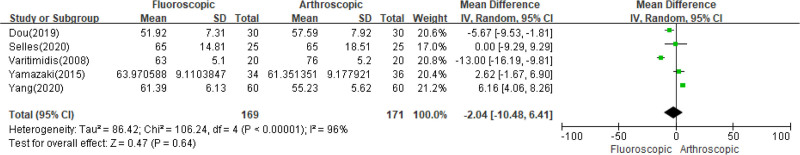
Comparison of range of flexion at the wrist joint between FAORIF and AAORIF group. AAORIF = arthroscopic-assisted open reduction and internal fixation, FAORIF = fluoroscopic-assisted open reduction and internal fixation.

Five studies^[19–23]^ reported that the ROM of flexion of the wrist and significant heterogeneity (*I*^2^ = 96%; *P* < .0001) were detected among the studies, and the meta-analysis of the combined effect sizes for the outcome yielded nonsignificant results(mean difference = ‐2.04, 95% confidence interval ‐10.48 to 6.48, *P* = .64), as illustrated in Figure 2. A sensitivity analysis was conducted. However, no study was found to have a significant impact on the results (Fig. 2).

#### 3.3.2. Pronation

**Figure 3.**
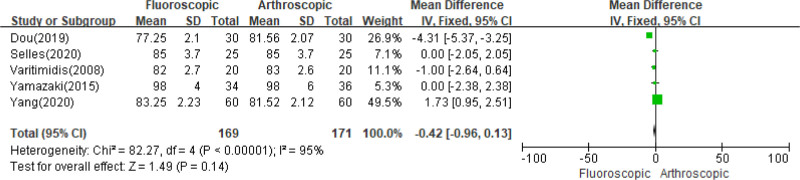
Comparison of range of pronation at the wrist joint between FAORIF and AAORIF group. AAORIF = arthroscopic-assisted open reduction and internal fixation, FAORIF = fluoroscopic-assisted open reduction and internal fixation.

Five trials^[19–23]^ included 340 cases that discussed the ROM of pronation of the wrist joint, and the overall estimate indicated a substantial presence of heterogeneity (*I*^2^ = 95%; *P* < .00001). No significant difference in pronation was found between the 2 groups at the final follow-up (MD = ‐0.42, 95% CI: ‐0.96, 0.13; *P* = .14) (Fig. 3). A sensitivity analysis was conducted and no study was found to have a significant impact on the results (Fig. 3).

#### 3.3.3. Extension

**Figure 4.**
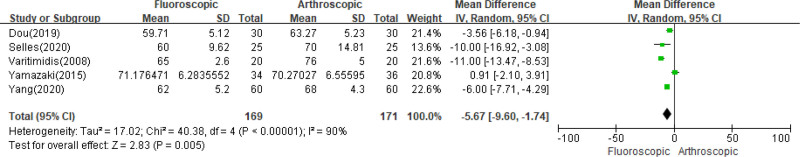
Comparison of range of extension at the wrist joint between FAORIF and AAORIF group. AAORIF = arthroscopic-assisted open reduction and internal fixation, FAORIF = fluoroscopic-assisted open reduction and internal fixation.

Dates from 5 studies^[19–23]^ were pooled for this variable, and the meta-analysis results indicated a significant pooled effect size for extension, with a favor towards the AAORIF group (*I*^2^ = 90%, *P* < .00001; MD = ‐5.67, 95% CI ‐9.60 to ‐1.74; *P* = .005), as shown in Figure 4. A sensitivity analysis was conducted and no study was found to have a significant impact on the results (Fig. 4).

#### 3.3.4. Supination

**Figure 5.**
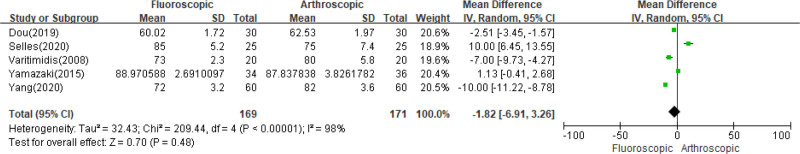
Comparison of range of supination at the wrist joint between FAORIF and AAORIF group. AAORIF = arthroscopic-assisted open reduction and internal fixation, FAORIF = fluoroscopic-assisted open reduction and internal fixation.

A total of 5 literatures^[19–23]^ with 340 patients reported the supination wrist joint. In the pooled analysis, there were no statistically significant differences in supination between the 2 groups (*I*^2^ = 98%, *P* < .00001; MD = ‐1.82, 95% CI ‐6.91 to 3.26; *P* = .48), as shown in Figure 5. We conducted a sensitivity analysis and found no studies that significantly affected the results (Fig. 5).

### 3.4. Complications

**Figure 6.**
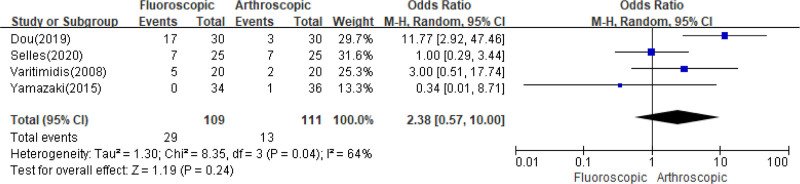
Comparison of the rate of complications between FAORIF and AAORIF group. AAORIF = arthroscopic-assisted open reduction and internal fixation, FAORIF = fluoroscopic-assisted open reduction and internal fixation.

The incidence of complications was reported by 4 studies^[19–22]^ with 220 subjects, on pooled analysis, there were no statistically significant differences in the complication rates among the 2 treatment (*I*^2^ = 64%, *P* = .04; OR = 2.38; 95% CI = 0.57–10.00; *P* = .24) (shown in Fig. 6). A sensitivity analysis was conducted and no study was found to have a significant influence on the results (Fig. 6).

### 3.5. Visual analog scale

**Figure 7.**
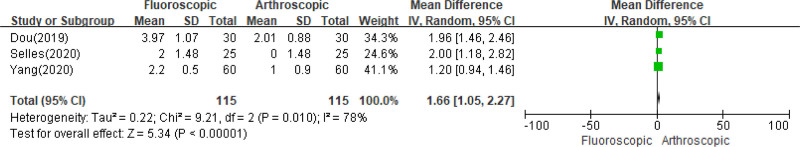
Comparison of the VAS score between FAORIF and AAORIF group. AAORIF = arthroscopic-assisted open reduction and internal fixation, FAORIF = fluoroscopic-assisted open reduction and internal fixation, VAS = visual analog scale.

The intensity of pain was measured on a VAS scale of 0 to 10, with a lower score representing a better condition; data regarding VAS at the final follow-up were available in 3 articles^[19,20,23]^ with 115 patients in the FAORIF group and the leftover patients in the AAORIF group; the statistical results demonstrated significant heterogeneity among the 3 studies (*I*^2^ = 78%; *P* = .010), and the overall estimate showed that the VAS was significantly lower in patients receiving AAORIF than in the FAORIF group (MD = 1.66, 95% CI = 1.05–2.27; *P* < .48) (shown in Fig. 7). A sensitivity analysis was conducted and no study was found to significantly impact the results (Fig. 7).

### 3.6. Operative time

**Figure 8.**
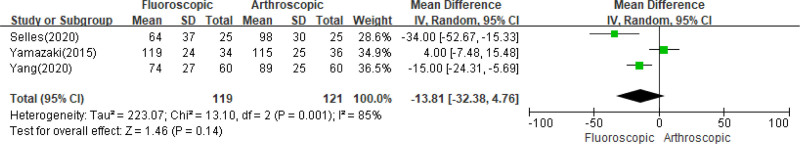
Comparison of the operative time between FAORIF and AAORIF group. AAORIF = arthroscopic-assisted open reduction and internal fixation, FAORIF = fluoroscopic-assisted open reduction and internal fixation.

Comparison of operative time was reported in only 3 articles,^[20,22,23]^ which enrolled 121 patients treated for AAORIF and 119 patients treated for FAORIF. According; however, there was no significant difference in operative time between the 2 surgical groups (*I*^2^ = 85%, *P* = .01; MD = ‐13.81, 95% CI = ‐32.38 to 4.76, *P* = .14), as depicted in Figure 8. We conducted a sensitivity analysis and found no studies that significantly influenced the results (Fig. 8).

### 3.7. DASH scores

**Figure 9.**
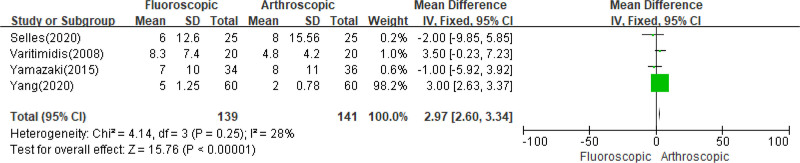
Comparison of the DASH scores between FAORIF and AAORIF group. AAORIF = arthroscopic-assisted open reduction and internal fixation, DASH = disability of arm, shoulder, and hand, FAORIF = fluoroscopic-assisted open reduction and internal fixation.

Data related to DASH scores at the final follow-up were available in 4 articles.^[20–23]^ The combined analysis revealed a significant decrease in DASH scores for patients who received AAORIF compared to those who received FAORIF (*I*^2^ = 28%, *P* = .25; MD = 2.97, 95% CI = 2.60–3.34, *P* < .00001) (Fig. 9).

## 4. Discussion

The ORIF technique has been commonly used as a conventional treatment option for distal radius fractures, especially intraarticular fractures.^[26]^ Compared to nonoperative treatment, this could result in a faster resumption of function in the first 3 to 6 months.^[27,28]^ Fluoroscopy has been frequently utilized to quickly and easily evaluate the reduction of the articular surface, showcasing its technical ease. Over the past ten years, there has been an increase in the use of fluoroscopic-assisted open reduction and internal fixation for distal radius fractures, and it has been employed as a practical and more accessible technology for evaluating the reduction of the articular surface.^[29,30]^ However, due to the inaccurate assessment of a 2 mm articular step-off or gap using direct fluoroscopy visualization, some patients may experience ongoing pain and stiffness in their wrist after surgery, leading to the development of traumatic arthritis.^[31,32]^ On the other hand, too many repeated C-arm fluoroscopies expose the doctor and the patient to a large amount of X-rays.

Arthroscopy can be used to assess screw protrusion, which can be adjusted accordingly.^[17,33]^ In particular, rotation of fracture fragments, which is difficult to judge under fluoroscopy, may be detected and corrected. Irrigation to remove fracture hematoma and debris potentially reduces the inflammatory reaction and improves the range of movement, and therapeutic treatment of intraarticular fractures of the distal radius under surveillance of wrist arthroscopy has attracted increasing attention from scholars with the expectation of better recovery.^[8,9,15,34]^ AAORIF was believed to provide better fracture reduction assistance to help reduce the incidence of traumatic arthritis. Some scholars found that AAORIF had superiority for treatment of distal radius fracture compared with FAORIF through the research. Nevertheless, the outcomes can vary. Certain researchers have discovered that fluoroscopic reduction with a top-notch image intensifier can be on par with arthroscopic reduction.^[21,22]^

Meta-analysis has been recognized as an effective method to resolve a wide variety of clinical questions by summarizing and reviewing published quantitative studies. As a result, this meta-analysis was conducted to provide a comprehensive comparison between AAORIF and FAORIF for the management of distal radius fracture. Wrist range of motion was one of the main outcomes measured in our study. The meta-analysis of the combined findings revealed a significant statistical advantage for wrist extension in the AAORIF group, while no differences were observed in flexion, pronation, and supination between the 2 groups. AAORIF has advantages like more accurate than fluoroscopy in assessing articular step-off or gap, affords the opportunity to identify and subsequently address associated soft tissue injuries.^[19,20]^ However, in contrast, other studies^[15,18]^ failed to observe any improvement in wrist range of motion among individuals who underwent arthroscopy for hematoma evacuation during internal fixation for distal radius fractures.

The DASH questionnaire assesses the level of difficulty in engaging in physical activities, the severity of symptoms, and the impact of the health issue on the patient’s daily functioning. In our study, another important outcome was the assessment of VAS scores and DASH scores, the pooled outcomes in this study suggested lower DASH scores and VAS scores in subjects receiving AAORIF in comparison with FAORIF. The meta-analysis by Shihab^[35]^ reported significantly better DASH scores in patients undergoing AAORIF for distal radius fractures. Varitimidis^[21]^ also described that the AAORIF group had less pain and an earlier return to daily activities than the FAORIF group at 12 month follow-up. During arthroscopy, we deduced that the assessment of reduction quality and the identification of associated ligament injuries could play a role in avoiding post-traumatic arthritis following distal radius fracture. The effectiveness of reconstructing soft tissues, such as carpal ligaments and the TFCC, might be linked to positive outcomes that lead to reductions in DASH scores and VAS scores.^[36,37]^

In our combined analysis, no significant differences were found in the risk of complications and operative time between the 2 groups, which contradicts the conjecture that arthroscopically assisted reduction could reduce the risk of posttraumatic osteoarthritis. We thought these results were limited by small numbers and variability in the time frames of outcome reporting.

This study has some limitations: only 5 of the included studies were RCTs, highlighting the difficulty of performing these studies in a clinical setting all, some of the included RCTs lacked the description of random methods, allocation hiding, and the implementation of blinding methods, and there is a high possibility of selection bias; second, the follow-up time points of the included studies were different, which may lead to reporting bias; however, this study only analyzed postoperative functional recovery in a general manner, without subgroup analysis at different time points. Furthermore, the exclusion of subgroups in the analysis was due to the impact of sample size and the varying fracture types and degrees observed in the studies included. For health economics and patient burden, there was no relevant evaluation in this study, which needs to be further evaluated in future studies.

## 5. Conclusion

In the surgical treatment of distal radius fractures, AAORIF might be associated with improved wrist extension and better DASH and VAS scores; however, the conclusion of this study must be verified by additional multicenter RCTs and high-quality studies, as the quality and quantity of the included studies are a limiting factor.

## Author contributions

**Conceptualization:** Wang Xiang.

**Formal analysis:** Shun Yang.

**Software:** Yabo Cheng.

**Writing – original draft:** Keyi Chen.
